# Association of dietary creatine intake from meat protein sources with different types of intestinal problems: insights from NHANES 2005–2010

**DOI:** 10.3389/fnut.2025.1586569

**Published:** 2025-07-25

**Authors:** Baohua Zheng, Zhicheng Huang, Zhiwei Wang, Junhao Du, Junhui Jiang, Chunhong Xiao, Yu Wang

**Affiliations:** ^1^Department of General Surgery, Fuzong Teaching Hospital of Fujian University of Traditional Chinese Medicine (900th Hospital), Fuzhou, China; ^2^Department of General Surgery, Dongfang Hospital of Xiamen University, School of Medicine, Xiamen University, Fuzhou, China; ^3^Department of General Surgery, Fuzong Clinical Medical College of Fujian Medical University, Fuzhou, China

**Keywords:** creatine, gut health, constipation, diarrhea, nutrition, NHANES

## Abstract

**Background:**

Given the growing interest in gut health, particularly in the context of irritable bowel syndrome (IBS), this study investigates the potential effects of dietary creatine intake on measures of gut health. Additionally, in response to anecdotal reports on the internet that have not been corroborated by clinical trials, this research examines the relationship between creatine consumption and gastrointestinal outcomes, aiming to address the existing knowledge gap in this area.

**Methods:**

This study utilized data from the 2005–2010 National Health and Nutrition Examination Survey (NHANES). Multivariable logistic regression and subgroup analyses were conducted to examine the association between dietary creatine intake and the risk of diarrhea and constipation. Additionally, restricted cubic spline (RCS) analysis was employed to assess potential nonlinear relationships.

**Results:**

In the fully adjusted model, each one-unit increase in the log-transformed 2-day average dietary creatine intake—equivalent to a tenfold increase in absolute intake—was associated with a 19% lower risk of chronic constipation (adjusted OR = 0.81, 95% CI: 0.65–0.96, *p* = 0.015). However, no significant association was found between dietary creatine intake and chronic diarrhea (adjusted OR = 1.04, 95% CI: 0.87–1.36, *p* = 0.421). The protective effect of higher dietary creatine intake against chronic constipation was more pronounced in males (adjusted OR = 0.77, 95% CI: 0.66–0.89, *p* < 0.001), younger individuals (adjusted OR = 0.89, 95% CI: 0.79 ∼ 0.99, *p* = 0.047)), and participants without cardiovascular disease (adjusted OR = 0.91, 95% CI: 0.83 ∼ 0.99, *p* = 0.047). RCS analysis confirmed a linear relationship between 2-day average dietary creatine intake and the risk of chronic constipation after adjusting for confounding variables.

**Conclusion:**

Higher dietary creatine intake may offer protective benefits against chronic constipation, particularly in specific subgroups, while showing no significant association with chronic diarrhea. Further large-scale studies are warranted to clarify creatine’s role in gastrointestinal health. These findings highlight the potential of creatine as a dietary factor in promoting gut health.

## Introduction

Chronic diarrhea and constipation are prevalent gastrointestinal disorders that significantly impact global quality of life. Chronic diarrhea is estimated to affect 4–5% of the global population, while chronic constipation affects 10–15% of adults (1, 2). These conditions are influenced by a range of factors, including diet, age, gender, physical activity, and underlying conditions such as irritable bowel syndrome (IBS), inflammatory bowel disease (IBD), and metabolic disorders(3, 4). Epidemiological studies have shown that the incidence of chronic constipation increases with age, especially among women, while chronic diarrhea is often associated with infections, inflammation, and certain medications (5, 6). Despite their high prevalence, effective treatments for these disorders remain limited, highlighting the need for further research into the potential impact of dietary factors.

Creatine, a naturally occurring compound primarily found in muscle tissue, plays a vital role in energy metabolism, particularly in the regeneration of adenosine triphosphate (ATP). Synthesized in the liver, kidneys, and pancreas from the amino acids glycine, arginine, and methionine, creatine is also acquired through dietary sources, such as red meat and fish (7, 8). On average, adults produce 1–2 g of creatine daily, with an additional 1–2 g obtained from their diet (9). While creatine is widely known for its role in muscle energy metabolism, emerging research is exploring its therapeutic potential in various diseases, including neurodegenerative disorders (e.g., Parkinson’s and Alzheimer’s) and cardiovascular diseases (10, 11). However, its effects on gastrointestinal health remain underexplored, with limited research on its impact on gut function.

Although creatine has been shown to affect various physiological systems, evidence regarding its influence on gastrointestinal motility and function is insufficient. Some studies suggest that creatine supplementation may alter gut microbiota composition or influence gastrointestinal motility, potentially affecting conditions such as chronic diarrhea and constipation (12, 13). However, the underlying mechanisms remain unclear, and there is currently no consensus on whether creatine intake exacerbates or alleviates these conditions. This knowledge gap underscores the importance of investigating the role of dietary creatine in chronic gastrointestinal diseases.

Given the limited research on the relationship between creatine and chronic gastrointestinal diseases, this study utilizes data from the National Health and Nutrition Examination Survey (NHANES) to explore the association between 2-day average dietary creatine intake and the risk of chronic diarrhea and constipation in United States adults aged 20 and older. The study also examines how demographic factors such as age, gender, and physical activity, along with chronic diseases like diabetes and hypertension, influence the relationship between dietary creatine intake and gastrointestinal function. Ultimately, this study aims to improve understanding of the potential dietary impacts on chronic bowel habits and provide new dietary recommendations for individuals affected by these conditions.

## Materials and methods

### Study population

**FIGURE 1:**
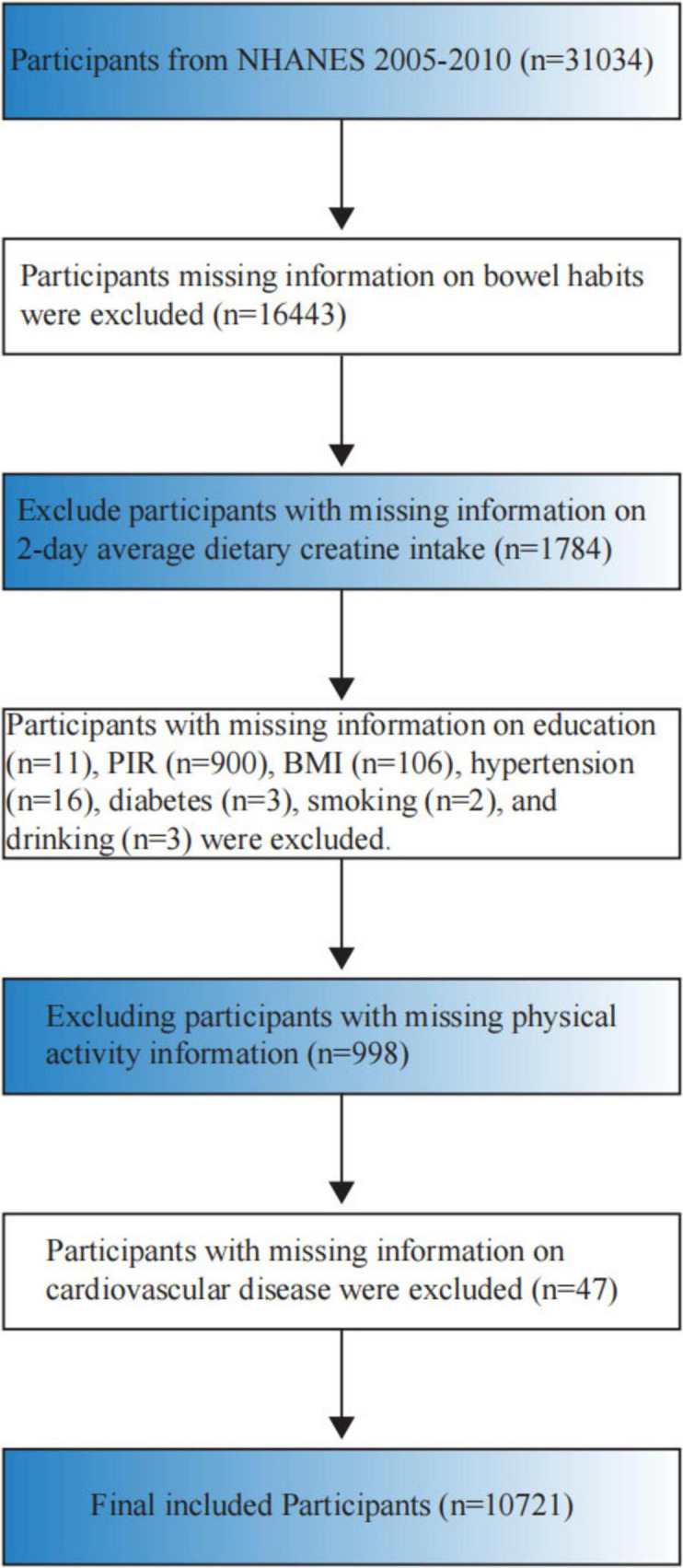
Flowchart of study participant inclusion and exclusion criteria.

The National Health and Nutrition Examination Survey (NHANES), administered by the National Center for Health Statistics (NCHS), assesses the health and nutritional status of the United States civilian, non-institutionalized population, including both adults and children. For this study, we used data from the 2005–2010 NHANES cycle, which included 31,034 participants. Strict exclusion criteria were applied, resulting in the exclusion of 20,313 participants who did not meet the criteria. Reasons for exclusion were as follows: (1) lack of information on bowel habits (*n* = 16,443); (2) absence of data on 2-day average dietary creatine intake (*n* = 1,784); (3) missing education information (*n* = 11); (4) missing family income-to-poverty ratio data (*n* = 900); (5) missing Body Mass Index (BMI) data (*n* = 106); (6) absence of hypertension status data (*n* = 16); (7) missing diabetes status data (*n* = 3); (8) lack of alcohol use data (*n* = 3); (9) absence of cardiovascular disease data (*n* = 47); (10) missing physical activity data (*n* = 998). After applying these exclusions, a total of 10,721 participants remained in the study (Figure 1).

### Assessment of 2-day average dietary creatine intake and bowel habits

Animal proteins, including meat, poultry, fish, and shellfish, are the primary dietary sources of creatine. The NHANES database collects participants’ 2-day dietary intake through the dietary interview questionnaire. This intake is recorded over two separate 24-hour periods, with the initial interview conducted in person during the Mobile Examination Center (MEC) assessment and a follow-up interview conducted by phone several days later. Using United States Department of Agriculture (USDA) food codes, the MyPyramid Equivalents database links food subcategories to the NHANES database, allowing the calculation of daily animal protein intake. The average creatine concentration from all animal protein sources was estimated at 0.11 g per ounce. The 2-day average dietary creatine intake was then calculated in grams and used as the primary exposure (14).

### Bowel health survey in the mobile examination center (MEC)

The Bristol Stool Form Scale (BSFS) is a key component of the bowel health questionnaire. Participants were shown an image with colored illustrations and descriptions of seven BSFS types and were asked to select the number that corresponded to their usual or most common stool type. Participants with Type 1 stools (separate hard lumps, like nuts) or Type 2 stools (sausage-shaped but lumpy) were classified as having chronic constipation, while those with Type 6 stools (fluffy pieces, ragged edges, mushy stool) or Type 7 stools (watery, no solid pieces) were classified as having chronic diarrhea. The remaining stool types were classified as normal bowel habits. Participants also recorded the frequency of their bowel movements by answering the question, “How often do you usually have bowel movements?”

### Cardiovascular disease and physical activity

Cardiovascular disease (CVD) status was determined using the “medical conditions questionnaire.” Participants were considered to have CVD if they answered “yes” to the question, “Have you ever been told you have congestive heart failure, coronary heart disease, angina, myocardial infarction, or stroke?”

Metabolic equivalents (METs) are commonly used to quantify energy expenditure during various activities. MET values were used to classify participants’ physical activity levels. The NHANES data utilized the Global Physical Activity (PA) questionnaire, allowing participants to self-report their physical activity. Physical activity (PA) was calculated using MET values, weekly exercise frequency, and duration. PA was classified as sedentary if PA < 600 MET-min/week and active if PA ≥ 600 MET-min/week. The following formula was used:


P⁢A⁢(MET-min/week)=M⁢E⁢T×D⁢u⁢r⁢a⁢t⁢i⁢o⁢n×W⁢e⁢e⁢k⁢l⁢y⁢f⁢r⁢e⁢q⁢u⁢e⁢n⁢c⁢y

### Covariates

The study collected participants’ sociodemographic characteristics and lifestyle factors as covariates, including age, sex, race/ethnicity (Mexican American, Other Hispanic, Non-Hispanic White, Non-Hispanic Black, Other Race), education level (< 9th grade, 9–11th grade, high school diploma/GED, some college/AA degree, ≥ college graduate), family income-to-poverty ratio, BMI, drinking status, smoking status, hypertension, and diabetes, Total calories, total protein, total dietary fiber, vitamin B1, vitamin B2, vitamin B6, vitamin B12, vitamin C, vitamin K, vitamin D. Hypertension was defined as “ever told you had high blood pressure” or “currently taking prescribed medication for high blood pressure (HBP).” Diabetes was defined as “doctor told you have diabetes,” “two-hour glucose oral glucose tolerance test (OGTT) (mmol/L) ≥ 11.1,” or “fasting glucose (mmol/L) ≥ 7.0.” Prediabetes was defined as “ever told you have prediabetes,” “two-hour glucose (OGTT) (mmol/L) = 7.8–11.1,” or “fasting glucose (mmol/L) = 6.1–6.9.” Smoking status was classified as non-smokers (never smoked or quit smoking for more than 1 year) and current smokers (currently smoking, smoked more than 1 day in the past 30 days, wake up to smoke, or smoke more than two cigarettes per day after quitting). Drinking status was classified as non-drinkers (drank fewer than 12 drinks in a lifetime) and current drinkers (drank at least 12 drinks in a year or more than 6 times in the past 12 months). BMI was calculated as weight (kg) divided by height (m) squared (14).

### Statistical analyses

Statistical analyses were conducted in accordance with the official NHANES guidelines. Final weights were determined based on the NHANES data analysis guidelines for the 2005–2010 cycle, with weighted data representing 13,556 United States citizens per sample. Continuous variables were described using means and standard deviations (SD), while categorical variables were presented as case counts and weighted percentages. Chi-square tests or Student’s *t-*tests were used to assess group differences. Multivariable logistic regression analysis was performed to investigate the relationship between 2-day average dietary creatine intake and IBD, adjusting for potential confounders. Model 1 was a univariate analysis without adjustments. Model 2 adjusted for covariates including age, sex, race/ethnicity, education, family income-to-poverty ratio, and BMI. Model 3 further adjusted for drinking status, smoking status, hypertension, and diabetes. Finally, Model 4 also adjusted for cardiovascular disease (CVD) and physical activity (PA),Total calories, total protein, total dietary fiber, vitamin B1, vitamin B2, vitamin B6, vitamin B12, vitamin C, vitamin K, vitamin D. Restricted cubic spline (RCS) analysis was conducted to examine the nonlinear relationship between dietary creatine intake and bowel habits, and to perform subgroup analyses and interaction tests to explore the relationship between creatine intake and the risk of chronic constipation or diarrhea in different populations. Missing data were imputed using multiple imputation to minimize sample size loss and reduce bias due to missing data. Data processing and analysis were conducted using IBM SPSS Statistics (version 24.0) and R software (version 4.3.0), with a two-sided *p*-value < 0.05 considered statistically significant.

## Results

### Participant characteristics based on different bowel habits

**FIGURE 2:**
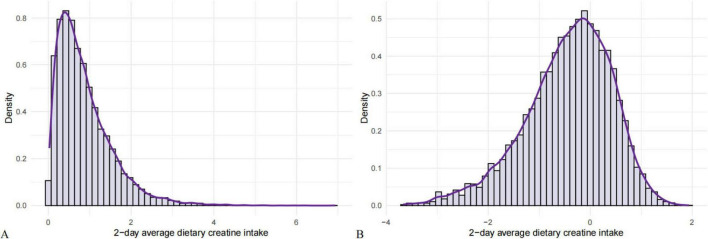
Normality test of 2-day average dietary creatine intake. **(A)** Original distribution of 2-day average dietary creatine intake. **(B)** Logarithmically transformed 2-day average dietary creatine intake.

We conducted a normality test on the distribution of 2-day average dietary creatine intake and found it to be skewed. Consequently, a logarithmic transformation was applied to normalize the distribution of 2-day average dietary creatine intake and mitigate the impact of skewness on the study results (Figure 2).

Table 1 presents the baseline characteristics of participants based on their bowel habits. This study included 10,721 participants from the NHANES 2005–2010 cycle, with a mean age of 49.29 years (SD = 17.65), consisting of 5,390 females (50.28%) and 5,331 males (49.72%). Of these, 787 participants had chronic constipation, with a mean age of 47.04 years (SD = 18.69), including 548 females (69.63%) and 239 males (30.37%). A total of 824 participants had chronic diarrhea, with a mean age of 53.28 years (SD = 16.36), including 463 females (56.19%) and 361 males (43.81%). Compared to healthy participants, those with chronic constipation were younger (mean age 47.04 vs. 49.13, *p* < 0.001), had a higher percentage of females (69.63% vs. 30.37%, *p* < 0.001), lower family income (mean 2.29 vs. 2.69, *p* < 0.001), lower BMI (mean 28.26 vs. 29.06, *p* < 0.001), were more likely to drink alcohol (81.70% vs. 18.30%, *p* < 0.001), had less hypertension (29.35% vs. 70.65%, *p* < 0.001), less diabetes (65.57% vs. 34.43%, *p* < 0.001), less cardiovascular disease (11.56% vs. 88.44%, *p* = 0.027), and were more physically active (50.44% vs. 40.05%, *p* < 0.001). Compared to healthy participants, those with chronic diarrhea were older (mean age 53.28 vs. 49.13, *p* < 0.001), had a higher percentage of females (56.19% vs. 43.81%, *p* < 0.001), lower family income (mean 2.30 vs. 2.69, *p* < 0.001), higher BMI (mean 30.59 vs. 29.06, *p* < 0.001), were more likely to drink alcohol (85.68% vs. 14.32%, *p* < 0.001), had less hypertension (55.95% vs. 44.05%, *p* < 0.001), less diabetes (59.95% vs. 34.44%, *p* < 0.001), less cardiovascular disease (12.86% vs. 87.14%, *p* = 0.027), and were more physically active (52.79% vs. 47.21%, *p* < 0.001). Regarding 2-day average dietary creatine intake, participants with abnormal bowel habits had lower intake compared to healthy participants (chronic constipation: mean 0.86 vs. 0.87; chronic diarrhea: mean 0.91 vs. 0.87; *p* < 0.001).

**TABLE 1:** Characteristics of adult participants with different bowel habits in NHANES (2005–2010) (*N* = 10721).

Characteristics	Total (*n* = 10721)	Normal (*n* = 9110)	Chronic constipation (*n* = 787)	Chronic diarrhea (*n* = 824)	*P*
Age(year)^a^	49.29 ± 17.65	49.13 ± 17.62	47.04 ± 18.69	53.28 ± 16.36	<0.001
Sex^b^					<0.001
Female	5390 (50.28)	4379 (48.07)	548 (69.63)	463 (56.19)	
Male	5331 (49.72)	4731 (51.93)	239 (30.37)	361 (43.81)	
Race/ethnicity^b^					<0.001
Mexican American	1798 (16.77)	1491 (16.37)	140 (17.79)	167 (20.27)	
Other Hispanic	836 (7.80)	681 (7.48)	83 (10.55)	72 (8.74)	
Non-Hispanic White	5582 (52.07)	4833 (53.05)	357 (45.36)	392 (47.57)	
Non-Hispanic Black	2087 (19.47)	1745 (19.15)	181 (23.00)	161 (19.54)	
Other Race	418 (3.90)	360 (3.95)	26 (3.30)	32 (3.88)	
Education^b^					<0.001
<9th grade	1051 (9.80)	805 (8.84)	93 (11.82)	153 (18.57)	
9–11th grade	1651 (15.40)	1349 (14.81)	155 (19.70)	147 (17.84)	
High school diploma/GED	2588 (24.14)	2175 (23.87)	211 (26.81)	202 (24.51)	
Some college/AA degree	3137 (29.26)	2748 (30.16)	198 (25.16)	191 (23.18)	
≥College graduate	2294 (21.40)	2033 (22.32)	130 (16.52)	131 (15.90)	
Family income to poverty ratio^a^	2.63 ± 1.62	2.69 ± 1.63	2.29 ± 1.55	2.30 ± 1.57	<0.001
BMI^a^	29.12 ± 6.70	29.06 ± 6.63	28.26 ± 6.72	30.59 ± 7.31	<0.001
BMI group^b^					<0.001
Normal	2857 (26.65)	2445 (26.84)	254 (32.27)	158 (19.17)	
Underweight	162 (1.51)	130 (1.43)	17 (2.16)	15 (1.82)	
Overweight	3688 (34.40)	3153 (34.61)	262 (33.29)	273 (33.13)	
Obesity	4014 (37.44)	3382 (37.12)	254 (32.27)	378 (45.87)	
Drinking status^b^					<0.001
No	1310 (12.22)	1048 (11.50)	144 (18.30)	118 (14.32)	
Yes	9411 (87.78)	8062 (88.50)	643 (81.70)	706 (85.68)	
Smoking status^b^					0.083
No	7737 (72.17)	6570 (72.12)	590 (74.97)	577 (70.02)	
Yes	2984 (27.83)	2540 (27.88)	197 (25.03)	247 (29.98)	
Hypertension^b^					<0.001
No	7004 (65.33)	5987 (65.72)	556 (70.65)	461 (55.95)	
Yes	3717 (34.67)	3123 (34.28)	231 (29.35)	363 (44.05)	
Diabetes^b^					<0.001
No	6880 (64.17)	5870 (64.43)	516 (65.57)	494 (59.95)	
Yes	1395 (13.01)	1139 (12.50)	100 (12.71)	156 (18.93)	
Borderline	2446 (22.82)	2101 (23.06)	171 (21.73)	174 (21.12)	
Cardiovascular disease^b^					0.027
No	9602 (89.56)	8188 (89.88)	696 (88.44)	718 (87.14)	
Yes	1119 (10.44)	922 (10.12)	91 (11.56)	106 (12.86)	
Physical activity					<0.001
No	4604 (42.94)	3825 (41.99)	390 (49.56)	389 (47.21)	
Yes	6117 (57.06)	5285 (58.01)	397 (50.44)	435 (52.79)	
Total calories^a^	2144.91 ± 1006.01	2171.33 ± 1018.15	1945.70 ± 897.44	2043.03 ± 940.16	<0.001
Total protein^a^	83.19 ± 43.05	84.26 ± 43.50	74.30 ± 37.21	79.81 ± 42.13	<0.001
Total dietary fiber^a^	16.13 ± 9.80	16.31 ± 9.77	14.49 ± 8.83	15.72 ± 10.78	<0.001
Vitamin B1^a^	1.62 ± 0.98	1.64 ± 0.99	1.47 ± 0.82	1.57 ± 0.96	<0.001
Vitamin B2^a^	2.16 ± 1.29	2.18 ± 1.29	1.95 ± 1.16	2.11 ± 1.37	<0.001
Vitamin B6^a^	2.03 ± 1.42	2.06 ± 1.43	1.81 ± 1.28	1.95 ± 1.43	<0.001
Vitamin B12^a^	5.49 ± 7.27	5.55 ± 7.35	4.75 ± 3.93	5.55 ± 8.66	0.013
Vitamin C^a^	87.56 ± 99.50	88.73 ± 100.07	81.04 ± 87.64	80.84 ± 103.35	0.015
Vitamin K^a^	97.67 ± 145.70	98.43 ± 144.07	85.00 ± 127.52	101.34 ± 176.32	0.035
Vitamin D^a^	4.73 ± 5.41	4.76 ± 5.42	4.73 ± 5.67	4.37 ± 5.02	0.209
Meat protein intake^a^	56.05 ± 5.80	56.05 ± 5.80	55.41 ± 5.15	58.63 ± 5.80	<0.001
2-day average dietary creatine intake^a^	0.87 ± 0.09	0.87 ± 0.09	0.86 ± 0.08	0.91 ± 0.09	<0.001
2-day average dietary creatine intake group^b^					<0.001
Q1	2680 (25.00)	2224 (24.41)	244 (31.00)	212 (25.73)	
Q2	2680 (25.00)	2263 (24.84)	209 (26.56)	208 (25.24)	
Q3	2680 (25.00)	2289 (25.13)	179 (22.74)	212 (25.73)	
Q4	2681 (25.01)	2334 (25.62)	155 (19.70)	192 (23.30)	

^a^ANOVA;

^b^Chi-square test; SD, standard deviation.

Regarding 2-day average dietary creatine intake, participants with abnormal bowel habits had lower intake compared to healthy participants (chronic constipation: mean 0.10 vs. 0.12; chronic diarrhea: mean 0.11 vs. 0.12; *p* = 0.240).

### Relationship between 2-day average dietary creatine intake and different bowel habits

**FIGURE 3:**
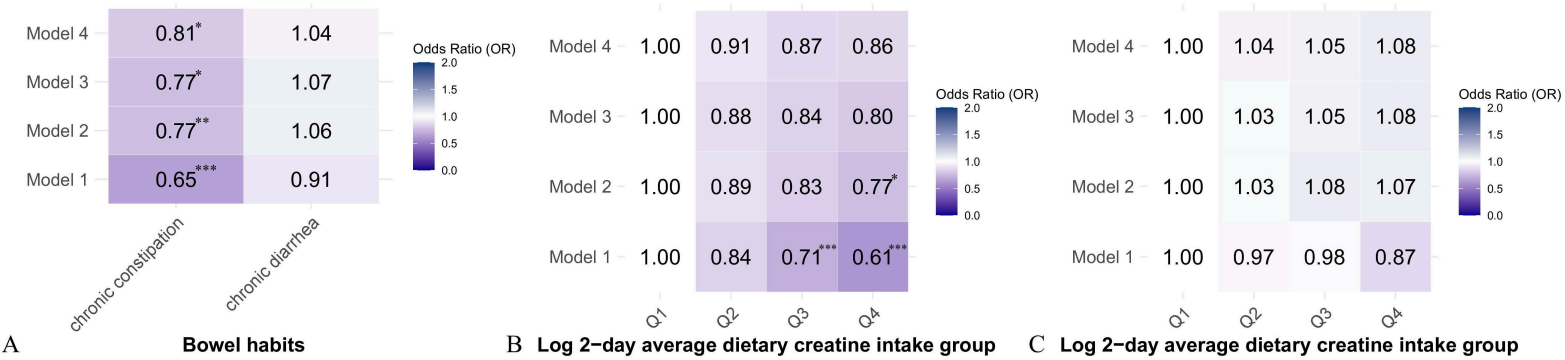
Heat map of the correlation between 2-day average dietary creatine intake and different types of intestinal problems. **(A)** OR values of continuous variables between 2-day average dietary creatine intake and different types of intestinal problems. **(B)** Quantile model of 2-day average dietary creatine intake and chronic constipation. **(C)** Quantile model of 2-day average dietary creatine intake and chronic diarrhea. *p < 0.05, **p < 0.01, ***p < 0.001.

Tables 2 and 3 show the relationship between 2-day average dietary creatine intake and the risk of chronic constipation and diarrhea, respectively. In the crude model, 2-day average dietary creatine intake was negatively associated with the risk of chronic constipation (OR = 0.65, 95% CI: 0.51 ∼ 0.75, *p* < 0.001). In Model 4, after adjusting for potential confounders such as sex, age, race/ethnicity, education, family income-to-poverty ratio, BMI, drinking status, smoking status, hypertension, diabetes, cardiovascular disease, and physical activity, the relationship between 2-day average dietary creatine intake and chronic constipation risk remained significantly negative (adjusted OR = 0.81, 95% CI: 0.65 ∼ 0.96, *p* = 0.015). Specifically, for each 1-unit increase in 2-day average dietary creatine intake, the risk of chronic constipation decreased by 19% (Figure 3A). Participants were divided into four groups based on the quartiles of 2-day average dietary creatine intake. In the unadjusted model, participants in the highest quartile had a 39% lower risk of chronic constipation compared to those in the lowest quartile (OR = 0.61, 95% CI: 0.49 ∼ 0.75, *p* < 0.001). After adjusting for confounders, the risk of chronic constipation remained negatively associated with 2-day average dietary creatine intake in each quartile compared to the lowest quartile, although this relationship was not statistically significant (second quartile: adjusted OR = 0.91, 95% CI: 0.74 ∼ 1.09, *p* = 0.105; third quartile: adjusted OR = 0.87, 95% CI: 0.70 ∼ 1.07, *p* = 0.154; fourth quartile: adjusted OR = 0.86, 95% CI: 0.68 ∼ 1.10, *p* = 0.184) (Figure 3B). Notably, trend tests indicated that, after adjusting for all relevant confounders, each increase in the quartile of 2-day average dietary creatine intake resulted in a 23% reduction in chronic constipation risk (adjusted OR = 0.82, 95% CI: 0.63 ∼ 0.98, *p* = 0.044) (Supplementary Table 1).

**TABLE 2:** Association between 2-day average dietary creatine intake and risk of chronic constipation in NHANES participants (2005–2010).

Variables	Model 1		Model 2		Model 3		Model 4	
	OR (95%CI)	*P*	OR (95%CI)	*P*	OR (95%CI)	*P*	OR (95%CI)	*P*
Log 2-day average dietary creatine intake	0.65 (0.51 ∼ 0.75)	<0.001	0.77 (0.64 ∼ 0.92)	0.002	0.77 (0.64 ∼ 0.93)	0.014	0.81 (0.65 ∼ 0.96)	0.015
**Log 2-day average dietary creatine intake group**								
Q1	1.00 (Reference)		1.00 (Reference)		1.00 (Reference)		1.00 (Reference)	
Q2	0.84 (0.69 ∼ 1.02)	0.081	0.89 (0.73 ∼ 1.08)	0.231	0.88 (0.73 ∼ 1.09)	0.212	0.91 (0.74 ∼ 1.09)	0.105
Q3	0.71 (0.58 ∼ 0.87)	<0.001	0.83 (0.67 ∼ 1.01)	0.056	0.84 (0.68 ∼ 1.02)	0.060	0.87 (0.70 ∼ 1.07)	0.154
Q4	0.61 (0.49 ∼ 0.75)	<0.001	0.77 (0.64 ∼ 0.98)	0.037	0.80 (0.65 ∼ 1.00)	0.055	0.86 (0.68 ∼ 1.10)	0.184
*P* for trend	<0.001		0.020		0.030		0.044	

OR, odds ratio; CI, confidence interval. Model 1: Crude. Model 2: adjust: sex, age, race/ethnicity, education, family income to poverty ratio, BMI. Model 3: adjust: sex, age, race/ethnicity, education, family income to poverty ratio, BMI, drinking status, smoking status, hypertension, diabetes. Model 4: adjust: sex, age, race/ethnicity, education, family income to poverty ratio, BMI, drinking status, smoking status, hypertension, diabetes, cardiovascular disease, physical activity, total calories, total protein, total dietary fiber, vitamin B1, vitamin B2, vitamin B6, vitamin B12, vitamin C, vitamin K, vitamin D.

**TABLE 3:** Association between 2-day average dietary creatine intake and risk of chronic diarrhea in NHANES participants (2005–2010).

Variables	Model 1		Model 2		Model 3		Model 4	
	OR (95%CI)	*P*	OR (95%CI)	*P*	OR (95%CI)	*P*	OR (95%CI)	*P*
Log 2-day average dietary creatine intake	0.91 (0.75 ∼ 1.10)	0.310	1.06 (0.86 ∼ 1.30)	0.214	1.07 (0.87 ∼ 1.21)	0.150	1.04 (0.87 ∼ 1.36)	0.421
**Log 2-day average dietary creatine intake group**								
Q1	1.00 (Reference)		1.00 (Reference)		1.00 (Reference)		1.00 (Reference)	
Q2	0.97 (0.80 ∼ 1.19)	0.792	1.03 (0.83 ∼ 1.24)	0.412	1.03 (0.82 ∼ 1.28)	0.542	1.04 (0.86 ∼ 1.27)	0.560
Q3	0.98 (0.80 ∼ 1.19)	0.813	1.08 (0.88 ∼ 1.34)	0.120	1.05 (0.85 ∼ 1.25)	0.521	1.05 (0.85 ∼ 1.39)	0.514
Q4	0.87 (0.71 ∼ 1.06)	0.170	1.07 (0.80 ∼ 1.34)	0.168	1.08 (0.80 ∼ 1.21)	0.642	1.08 (0.80 ∼ 1.35)	0.210
P for trend	0.213		0.539		0.607		0.618	

OR, odds ratio; CI: confidence interval. Model 1: Crude. Model 2: adjust: sex, age, race/ethnicity, education, family income to poverty ratio, BMI. Model 3: adjust: sex, age, race/ethnicity, education, family income to poverty ratio, BMI, drinking status, smoking status, hypertension, diabetes. Model 4: adjust: sex, age, race/ethnicity, education, family income to poverty ratio, BMI, drinking status, smoking status, hypertension, diabetes, cardiovascular disease, physical activity, total calories, total protein, total dietary fiber, vitamin B1, vitamin B2, vitamin B6, vitamin B12, vitamin C, vitamin K, vitamin D.

After adjusting for potential confounders, 2-day average dietary creatine intake was positively associated with the risk of chronic diarrhea. Specifically, for each 1-unit increase in 2-day average dietary creatine intake, the risk of chronic diarrhea increased by approximately 4%. However, this relationship was not statistically significant (adjusted OR = 1.04, 95% CI: 0.87 ∼ 1.36, *p* = 0.421) (Figure 3A and Table 3). Participants were divided into four groups based on quartiles of 2-day average dietary creatine intake. After adjusting for relevant confounders, compared to the lowest quartile, the risk of chronic diarrhea was positively associated with dietary creatine intake in the other quartiles (second quartile: adjusted OR = 1.04, 95% CI: 0.86 ∼ 1.27, *p* = 0.560; third quartile: adjusted OR = 1.05, 95% CI: 0.85 ∼ 1.39, *p* = 0.514; fourth quartile: adjusted OR = 1.08, 95% CI: 0.80 ∼ 1.35, *p* = 0.210) (Figure 3C). Trend test analysis indicated that for each 1-quartile increase in 2-day average dietary creatine intake, the risk of chronic diarrhea increased by 7%, although this relationship was not statistically significant (adjusted OR = 1.07, 95% CI: 0.82 ∼ 1.40, *p* = 0.618) (Supplementary Table 2).

### Subgroup analysis of the relationship between 2-day average dietary creatine intake and different bowel habits

**FIGURE 4:**
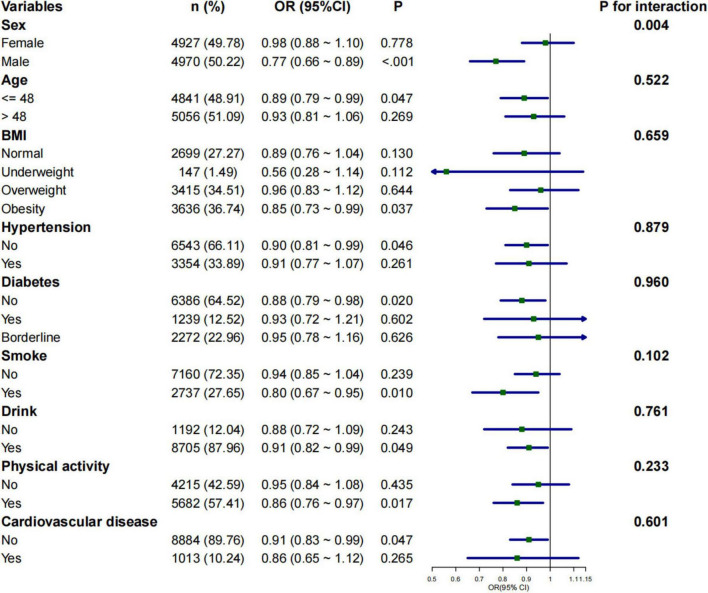
Subgroup analysis of the relationship between 2-day average dietary creatine intake and the risk of chronic constipation.

**FIGURE 5:**
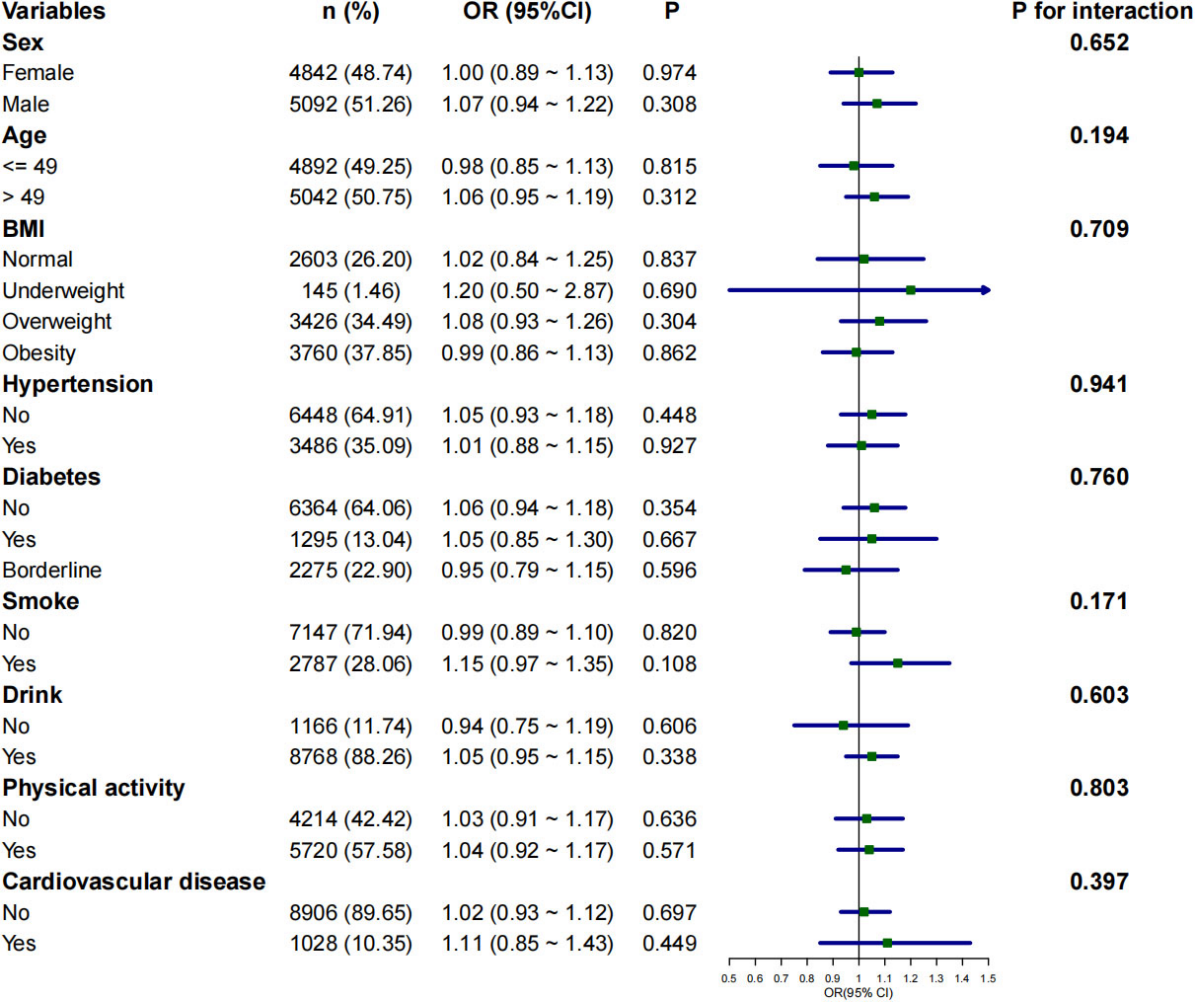
Subgroup analysis of the relationship between 2-day average dietary creatine intake and the risk of chronic diarrhea.

Subgroup analyses were conducted to assess whether the relationship between 2-day average dietary creatine intake and the risk of chronic constipation and diarrhea varied across different populations. The negative correlation between 2-day average dietary creatine intake and the risk of chronic constipation was consistent in male participants (adjusted OR = 0.77, 95% CI: 0.66 ∼ 0.89, *p* < 0.001), participants younger than 48 years (adjusted OR = 0.89, 95% CI: 0.79 ∼ 0.99, *p* = 0.047), obese participants (adjusted OR = 0.85, 95% CI: 0.73 ∼ 0.99, *p* = 0.037), participants without hypertension (adjusted OR = 0.90, 95% CI: 0.81 ∼ 0.99, *p* = 0.046), participants without diabetes (adjusted OR = 0.88, 95% CI: 0.79 ∼ 0.98, *p* = 0.020), smokers (adjusted OR = 0.80, 95% CI: 0.67 ∼ 0.95, *p* = 0.010), drinkers (adjusted OR = 0.91, 95% CI: 0.82∼ 0.99, *p* = 0.049), physically active individuals (adjusted OR = 0.86, 95% CI: 0.76 ∼ 0.97, *p* = 0.017), and participants without cardiovascular disease (adjusted OR = 0.91, 95% CI: 0.83 ∼ 0.99, *p* = 0.047) (Figure 4). The relationship between 2-day average dietary creatine intake and the risk of chronic diarrhea was not statistically significant across subgroups (Figure 5). Interaction tests suggested that gender modified the relationship between 2-day average dietary creatine intake and the risk of chronic constipation (P for interaction = 0.010). No significant interactions were observed between 2-day average dietary creatine intake and chronic diarrhea risk with factors such as gender, age, and others (Figures 4, 5).

### Linear and nonlinear relationships between 2-day average dietary creatine intake and different bowel habits

**FIGURE 6:**
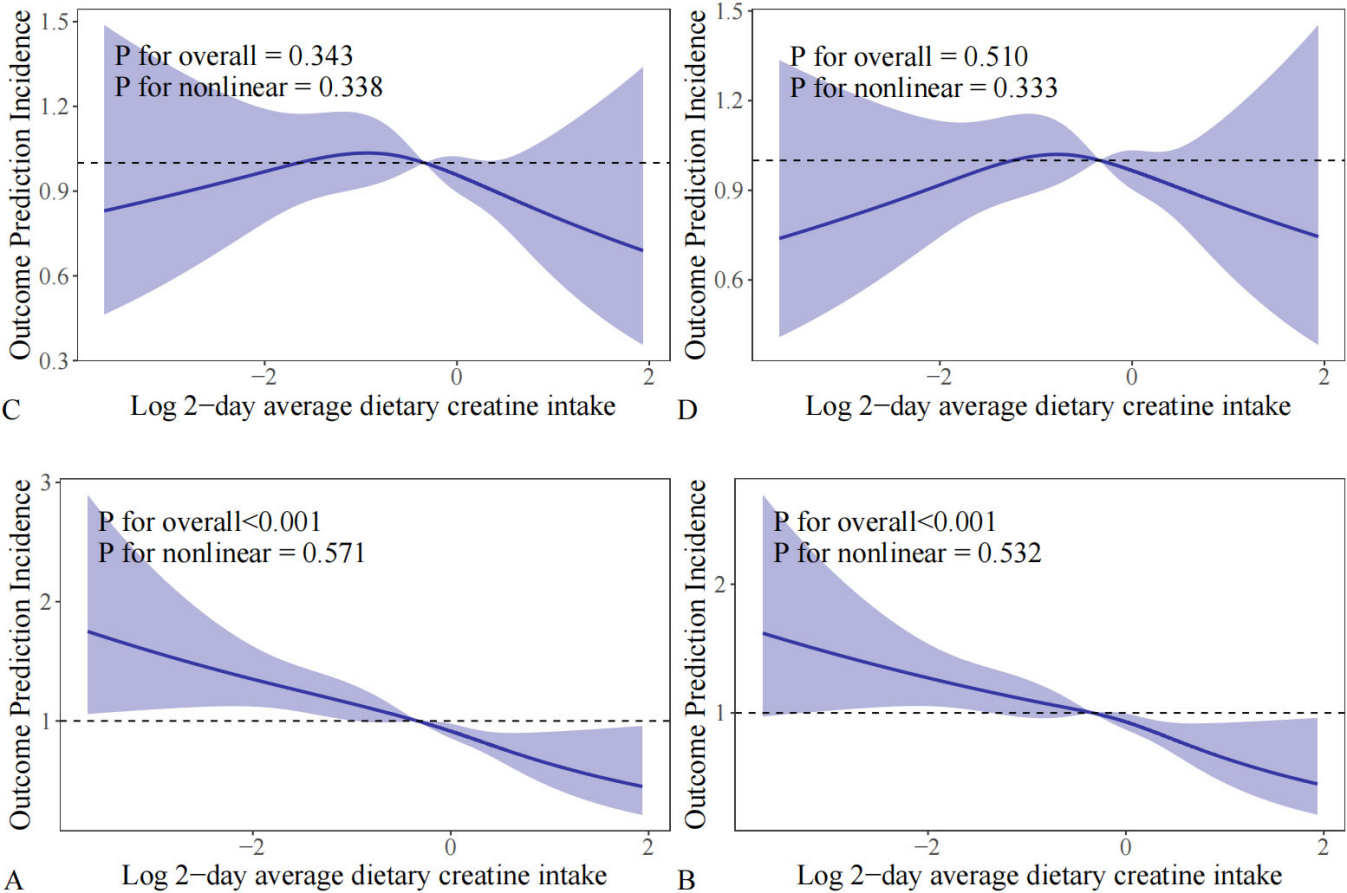
RCS analysis between 2-day average dietary creatine intake and different intestinal health problems. **(A)** Chronic constipation without adjustment; **(B)** chronic diarrhea without adjustment; **(C)** chronic constipation with adjustment; **(D)** chronic diarrhea with adjustment.

RCS analysis was performed to visually depict the changing relationship between 2-day average dietary creatine intake and different bowel habits. Before adjusting for potential confounders, the relationship between 2-day average dietary creatine intake and chronic constipation and diarrhea risk showed a linear pattern (chronic constipation: p for nonlinear = 0.571 and p for overall < 0.001; chronic diarrhea: p for nonlinear = 0.532 and p for overall < 0.001) (Figures 6A, B). After adjusting for potential confounders, the linear relationship between 2-day average dietary creatine intake and chronic constipation and diarrhea risk remained stable (chronic constipation: p for nonlinear = 0.343 and p for overall = 0.338; chronic diarrhea: p for nonlinear = 0.510 and p for overall = 0.333) (Figures 6C, D).

## Discussion

This study utilized data from 10,721 participants in the NHANES 2005–2010 cycle to investigate the association between dietary creatine intake and bowel habits, particularly chronic diarrhea and chronic constipation. The results revealed a significant negative correlation between dietary creatine intake and the risk of chronic constipation, with an adjusted odds ratio (OR) of 0.81, suggesting that higher creatine intake is associated with a lower likelihood of chronic constipation. In contrast, no statistically significant association was found between creatine intake and chronic diarrhea (adjusted OR = 1.07). Subgroup analyses indicated that the protective effect of creatine on constipation was more pronounced in males, younger individuals, smokers, drinkers, and those without certain comorbidities. Gender-specific interaction tests showed that the effect of creatine on constipation risk varied by gender, while age, BMI, and other factors did not exhibit significant interactions with creatine intake in relation to diarrhea.

Consistent with recent studies, this research underscores the beneficial effects of creatine supplementation on gastrointestinal health. Although creatine is primarily recognized for its muscle-enhancing properties, including promoting protein synthesis and supporting cell hydration by increasing water intake in muscle cells (15), gut cells also benefit from hydration, which may enhance material exchange and intestinal motility. A retrospective analysis involving 130 individuals found that patients with creatine transporter (CRTR) deficiency often experience weight gain and constipation (16). Animal experiments have shown that creatine supplementation in fish increases the expression of mRNA for CSAD, which leads to increased taurine production, thereby improving constipation through mechanisms such as regulating gut microbiota and altering stool bile acid composition. For instance, taurine supplementation in loperamide-induced constipated rats significantly increased the number of stool pellets (12, 17). Similarly, a controlled study in low-temperature organ storage found that creatine treatment improved gut mucosal barrier function and electrophysiology in rodent models compared to a control group after 10 h of storage (18). Additionally, creatine supplementation improved symptoms and endoscopic findings in a patient with Crohn’s disease, with ulceration and intestinal narrowing showing improvements (19). These findings suggest that creatine supplementation positively affects the intestinal barrier, which may contribute to its beneficial impact on gastrointestinal function.

However, our findings differ from studies suggesting that creatine intake may alleviate gastrointestinal discomfort, including diarrhea, in conditions like IBD (20, 21). The lack of a significant association with diarrhea in our study may be attributed to differences in creatine dosage, as higher doses in some studies may affect intestinal permeability (7). The observed differences could also be influenced by variations in participants’ lifestyle and dietary factors, such as exercise and ketogenic diets, which differ across studies. These findings suggest that lifestyle factors may moderate the relationship (22, 23).

An unexpected finding in our study is that creatine intake appears to have a significant protective effect on constipation in men and younger participants, but no similar association was found for chronic diarrhea. A meta-analysis on IBS found that constipation-predominant IBS is more common in women (40%) than in men (21%) (OR, 2.38; 95% CI, 1.45–3.92), while diarrhea-predominant IBS is more common in men (50%) than in women (31%) (OR, 0.45; 95% CI, 0.32–0.65), with no significant differences in mixed-type IBD (24). A study of 557 IBS patients (152 males) found that gastrointestinal discomfort was more common in females, who also experienced more severe symptoms such as bloating and hard stools (25). These gender-specific differences in gastrointestinal function suggest that females are more prone to constipation. Based on this, we hypothesize that creatine’s effects on gastrointestinal function may be influenced by differences in hormone levels or metabolic pathways. From a physiological perspective, creatine supplementation increases intramuscular creatine stores (including phosphocreatine and free creatine), which enhances muscle strength. Testosterone can increase muscle cell metabolic activity, promote creatine uptake, and enhance abdominal and pelvic floor muscle function, leading to more pronounced benefits in males (26). In a double-blind, placebo-controlled, crossover study, 20 young males underwent a 21-day creatine supplementation (or placebo) intervention, which showed that creatine supplementation may accelerate the conversion of testosterone to the more active dihydrotestosterone, enhancing its physiological effects (27). The interaction between gut microbiota and sex hormones has been an area of focus. In animal experiments, gonadectomy or postnatal overfeeding-induced gonadal hormone depletion made the gut microbiota more harmful. However, testosterone treatment suppressed these changes, improving gut barrier integrity (28–30). Recent research suggests that gender differences in gut microbiota may be influenced by bile acids, with males and females having significantly different bile acid pools (31). It is known that bile acids influence gut microbiota (32, 33), and recent studies further suggest that testosterone mediates gender-specific differences in gut microbiota composition through the bile acid signaling pathway (34). These interactions between creatine, sex hormones, and gut microbiota may explain why males benefit more from creatine intake in alleviating gastrointestinal discomfort.

Another unexpected finding in our study was the lack of a significant association between creatine intake and constipation risk in individuals with specific comorbidities, such as hypertension and diabetes. In a systematic review investigating the effects of creatine supplementation on patients with type 2 diabetes, findings indicate that compared to a placebo, creatine supplementation does not result in gastrointestinal-related adverse reactions (35, 36). Similar findings have been observed in hypertensive patients, where creatine improved cardiac energy utilization, reduced arrhythmia incidence, and enhanced myocardial function and cardiovascular health (37). The absence of a creatine effect on constipation in these groups may be due to vascular dysfunction associated with hypertension, which could affect gastrointestinal blood flow and gut microbiota. Additionally, diabetes patients often experience gastrointestinal complications, such as gastroparesis and constipation, which may limit creatine’s ability to improve gastrointestinal function (38–40).

This study contributes to the literature by indicating that dietary creatine may specifically protect against chronic constipation, particularly in subgroups such as males and younger individuals. These results support the hypothesis that creatine, in addition to its role in energy metabolism, may offer systemic benefits by promoting cell hydration and improving intestinal motility (15, 41). Our findings suggest that creatine could be part of dietary management for constipation, warranting further experimental studies on its role in gut health. Given that creatine’s effects vary by gender and age, future research should explore the underlying biological mechanisms, focusing on hormonal and metabolic factors to confirm these subgroup-specific findings (42).

The main limitation of this study is its cross-sectional design, which limits causal inference. Although we controlled for confounding variables, residual confounding may still exist, particularly given the complex effects of diet and lifestyle on gut health (43). Additionally, NHANES relies on self-reported dietary data, which may be subject to recall bias and inaccuracies. Another limitation is the lack of detailed data on the duration and dosage of creatine intake, which would provide a clearer understanding of its effects on bowel habits. These limitations highlight the need for more detailed longitudinal studies with dietary tracking to confirm and extend these findings.

## Conclusion

Overall, this study offers valuable insights into the potential role of dietary creatine in gut health. Given the significant inverse association between dietary creatine intake and chronic constipation, further research, including randomized controlled trials, is warranted to evaluate the potential benefits of creatine supplementation in managing chronic constipation.

## Data Availability

The datasets presented in this study can be found in online repositories. The names of the repository/repositories and accession number(s) can be found below: https://www.cdc.gov/nchs/nhanes/index.html.
