# Supplementary material for: Association of dietary creatine intake from meat protein sources with different types of intestinal problems: insights from NHANES 2005–2010
Source: Front Nutr. 2025 Jul 25;12:1586569. doi: 10.3389/fnut.2025.1586569 (PMC12331507; doi:10.3389/fnut.2025.1586569)
Supplement: Supplementary file 1 [file Data_Sheet_1.pdf]

Supplementary Table 1 Trend analysis between 2-day average dietary creatine intake and risk of chronic constipation

Variables	Model1		Model2		Model3		Model4	
	OR (95%CI)	*P*	OR (95%CI)	*P*	OR (95%CI)	*P*	OR (95%CI)	*P*
Trend	0.55 (0.43 ~ 0.69)	<.001	0.75 (0.58 ~ 0.96)	0.020	0.76 (0.59 ~ 0.97)	0.030	0.82 (0.63 ~ 0.98)	0.044

OR: Odds Ratio,CI: Confidence Interval

Model1: Crude

Model2: Adjust: Sex, Age, Race/ethnicity, Education, Family income to poverty ratio, BMI

Model3: Adjust: Sex, Age, Race/ethnicity, Education, Family income to poverty ratio, BMI, Drinking status, Smoking status, Hypertension, Diabetes

Model4: Adjust: Sex, Age, Race/ethnicity, Education, Family income to poverty ratio, BMI, Drinking status, Smoking status, Hypertension, Diabetes, Cardiovascular disease, Physical activity, Total calories, total protein, total dietary fiber, vitamin B1, vitamin B2, vitamin B6, vitamin B12, vitamin C, vitamin K, vitamin D

Supplementary Table 2 Trend analysis between 2-day average dietary creatine intake and risk of chronic diarrhea

Variables	Model1		Model2		Model3		Model4	
	OR (95%CI)	*P*	OR (95%CI)	*P*	OR (95%CI)	*P*	OR (95%CI)	*P*
Trend	0.86 (0.68 ~ 1.09)	0.212	1.08 (0.85 ~ 1.38)	0.539	1.07 (0.83 ~ 1.36)	0.607	1.07 (0.82 ~ 1.40)	0.618

OR: Odds Ratio,CI: Confidence Interval

Model1: Crude

Model2: Adjust: Sex, Age, Race/ethnicity, Education, Family income to poverty ratio, BMI

Model3: Adjust: Sex, Age, Race/ethnicity, Education, Family income to poverty

Variables	Model1		Model2		Model3		Model4	
	OR (95%CI)	*P*	OR (95%CI)	*P*	OR (95%CI)	*P*	OR (95%CI)	*P*

ratio, BMI, Drinking status, Smoking status, Hypertension, Diabetes

Model4: Adjust: Sex, Age, Race/ethnicity, Education, Family income to poverty ratio, BMI, Drinking status, Smoking status, Hypertension, Diabetes, Cardiovascular disease, Physical activity, Total calories, total protein, total dietary fiber, vitamin B1, vitamin B2, vitamin B6, vitamin B12, vitamin C, vitamin K, vitamin D
